# Oral Octreotide Capsules and Paltusotine in Management of Acromegaly

**DOI:** 10.17925/EE.2023.20.1.3

**Published:** 2023-11-08

**Authors:** David S McLaren, Khyatisha Seejore, Julie Lynch, Robert D Murray

**Affiliations:** 1. Department of Endocrinology, Leeds Centre for Diabetes & Endocrinology, Leeds Teaching Hospital NHS Trust, Leeds, UK; 2. Division of Cardiovascular and Diabetes Research, Leeds Institute of Cardiovascular and Metabolic Medicine (LICAMM), University of Leeds, Leeds, UK

**Keywords:** Acromegaly, lanreotide, ligand, octreotide, paltusotine, somatostatin

## Abstract

Injectable somatostatin receptor ligands (iSRL) are the most frequently utilized medical therapy in patients with acromegaly; however, satisfaction rates are suboptimal. Injections can result in local erythema, discomfort and subcutaneous nodule formation, encompassed with the inconvenience of attending either primary or secondary care medical facilities for injections every 4 weeks. Some patients also note breakthrough of acromegaly-related symptoms towards the end of the injection cycle. To improve acceptance and ultimately improve wellbeing of these individuals, two oral SRLs, oral octreotide capsules (OOC) and paltusotine, have been developed. The OOC combines an enteric coating to allow delivery to the small intestines and a transient permeability enhancer to enable oral bioavailability. Comparable octreotide levels are obtained with twice-daily OOC and subcutaneous octreotide 100 µg. Phase III studies show OOC to maintain equivalent biochemical control in at least 60% of patients previously receiving a stable dose of iSRL. In longer-term studies, the response to OOC was durable up to 3 years. Paltusotine is a novel potent orally available non-peptidyl somatostatin receptor subtype-2 ligand. Studies in healthy volunteers show dose-dependent suppression of growth hormone-releasing hormone-induced growth hormone secretion and suppression of insulin-like growth factor-I (IGF-I) with repeat doses. In the recent phase II study, patients with acromegaly who were partial responders (IGF-I 1.0 – 2.5 x upper limit of normal) to monotherapy with iSRL when switched to once-daily paltusotine maintained control of IGF-I within 20% of baseline or lower in 87% after 13 weeks. Adverse events with both OOC and paltusotine were reflective of those recognized with iSRL and occurred at a similar frequency. OOC and paltusotine are well-received additions to the therapeutic armamentarium in medical therapy for the management of acromegaly; however, further data on efficacy, tumour control and shrinkage are required to allow positioning of this medication within the management algorithm for acromegaly.

Acromegaly is the clinical consequence of chronic excessive exposure of the tissues to growth hormone (GH) and its second messenger, insulin-l ike growth factor-I (IGF-I ). The excess GH secretion is almost exclusively secondary to a GH-secreting pituitary adenoma (somatotropinomas); however, excess GH also results from ectopic GH-releasing hormone (GHRH) secretion, usually from a neuroendocrine tumour, in around 1% of cases. Symptoms classically involve somatic changes including coarsening of the facial features and enlargement of the hands and the feet, headaches, visual field defects, hyperhidrosis, fatigue, sexual dysfunction and paraesthesia. Diagnosis is frequently delayed due to the insidious nature of the disease and absence of specific symptoms or signs in the early stages. Acromegaly is associated with a number of long-term consequences inclusive of hypopituitarism, obstructive sleep apnoea, cardiomyopathy, diabetes mellitus, colonic polyps, hypertension, osteoporosis, vertebral fractures and arthropathy. Importantly, acromegaly is also associated with a significantly elevated mortality rate relating to cardiovascular, respiratory, and more contentiously, malignant disease.^1,2^

Management is targeted at relief of the patient's symptoms; reduction in the bulk of the pituitary adenoma when there are mass-related symptoms; optimizing biochemical markers of disease activity (GH and IGF-I ); and reversal or prevention of progression of associated long-term complications and mortality.^3,4^ Strict control of GH levels to <1.0 µg/L in association with a normal age-adjusted IGF-I level has been shown to normalize mortality to that of the background population as well as slow progression, or even reverse some of the associated long-term complications.^2^

## Management of acromegaly

For most cases of acromegaly, the initial therapeutic intervention remains transsphenoidal surgery (TSS) to remove or debulk the pituitary tumour whilst aiming to preserve normal pituitary function. Microadenomas operated on in specialist centres achieve biochemical remission rates approaching 90%;^3^ however, outcomes are poorer for patients with macroadenomas or with cavernous sinus invasion, where remission is achieved in less than 50%.^3,5^ As the majority of somatotroph tumours are macroadenomas, overall remission rates from surgery are in the region of 40–65%.^4^ Thus, around 35–60% of patients who undergo surgery, as well as those for who surgery is contraindicated, will require further interventions, which are not infrequently multimodality.^4^ For those in this cohort who continue to have biochemically active disease, second-l ine treatment includes repeat surgery, medical therapy or radiotherapy. Repeat TSS can be offered to individuals where the tumour residuum is mostly intrasellar, with remission reported in just over 50% of patients.^6,7^ In line with the Endocrine Society clinical practice guidelines however, the majority of patients are managed second line with medical therapy.^3^ This clinical guideline recommends use of first-generation injectable somatostatin receptor ligands (iSRL) in the majority of patients; cabergoline in those with only minimal disease activity; and consideration of pegvisomant in more resistant cases.^3^ Long-acting first-generation iSRL therapy is therefore generally considered as the preferred option for individuals with persistent disease post-TSS.

**Table 1:** Overview of injectable somatostatin receptor ligands (iSRL) used in treatment of acromegaly and novel oral somatostatin receptor ligands

	SSTR subtype ligand	Route of administration	Dosage	Accepted use in acromegaly	Frequent side effects
**Octreotide LAR**	SSTR2 and 5	Deep IM injection	10–30 mg every 28 days	Primary therapy, second-line therapy, combination therapy, surgical pre-treatment	GI disturbances, cholelithiasis*, injection site discomfort.
**Lanreotide autogel**	SSTR2 and 5	Deep SC injection	60–120 mg every 28 days	Primary therapy, second-line therapy, combination therapy, surgical pre-treatment	GI disturbances, cholelithiasis*, injection site discomfort
**Pasireotide LAR**	SSTR1, 2, 3 and 5	Deep IM injection	40–60 mg every 28 days	Inadequate control with first-generation iSRL, Combination therapy.	GI disturbances, cholelithiasis*, injection site discomfort, hyperglycaemia, QT prolongation
**Oral octreotide capsules**	SSTR2 and 5	Oral	20–40 mg twice per day	Patients controlled on first-generation iSRL	GI disturbances, cholelithiasis†
**Paltusotine**	SSTR2	Oral	10–60 mg once daily	Patients controlled on first-generation iSRL‡	GI disturbances, cholelithiasis†

*Cholelithiasis may occur with long-term usage.

*†Cholelitiasis likely to occur but long-term data is not available to date.*

*‡Positioning of paltusotine in acromegaly algorithm yet to be fully established.*

*GI = gastrointestinal; IM = intramuscular; LAR = long-acting repeatable; SC = subcutaneous; SSTR = somatostatin receptor subtype.*

iSRLs have been available for the last 30 years. The two first-generation iSRL preparations widely available currently are octreotide long-acting repeatable (LAR) and lanreotide autogel (*Table 1*). Both these iSRLs act primarily through the somatostatin receptor subtype (SSTR) 2, with lesser affinity for SSTR-5. Efficacy of these two analogues is equivalent with biochemical control represented by a GH <2.5 µg/L and a normal IGF-I level in 30–40% of individuals.^8–11^ Notably, similar biochemical outcomes are observed whether the iSRLs are used as primary or secondary therapy following TSS.^12^ First-generation iSRL also have remarkable inhibitory effects on tumour growth to the extent that <2% of individuals show tumour enlargement during long-term treatment.^12,13^

In addition to the use of iSRLs to induce long-term biochemical control, pre-operative use has been proposed to improve symptoms and reverse co-morbidities, in the hope of reducing surgical risk. Furthermore, in GH-secreting macroadenoma, pre-operative use of iSRL induces significant tumour shrinkage in a proportion of patients, thereby providing the pituitary surgeon with a smaller and more defined target.^12^ Whether pre-operative use of iSRL leads to improved surgical outcomes remains contentious; however, the available data favour improved post-operative outcomes.^14^

A novel second-generation iSRL, pasireotide, has affinity for a wider spectrum of SSTR subtypes (SSTR1-3 and 5) and is of value in a proportion of individuals who fail to achieve biochemical control with first-generation ligands. Side effects for all iSRL are predominantly gastrointestinal, particularly abdominal cramps and loose stools, which diminishes over time and with repeated injections. Patients on iSRL long term are also at risk of developing cholelithiasis. Dysglycaemia in the form of hyper-and hypoglycaemia can occur, though is significantly more frequent with pasireotide.^15^

Where biochemical control of the disease is not achieved following TSS and first-generation iSRLs, options include: (1) escalation of the dose or reduction in time between iSRL injections, which can lead to control of GH and IGF-I in a subset of partial responders to iSRL, though effects are modest; (2) use of pasireotide; (3) addition of cabergoline to first-generation iSRL.^16^ A meta-analysis of five studies revealed that the addition of cabergoline normalized age-adjusted IGF-I in 52% of the cohort, and reduced mean GH levels from 7.4 ± 12.5 µg/L to 3.6 ± 3.8µg/L.^17^ A number of similar studies published since the aforementioned meta-analysis show normalization of IGF-I levels to be achieved in 30–48%.^4,18,19^ The probability of achieving target IGF-I levels was highest for those with only mild-moderate disease activity; or (4) consideration of pegvisomant either as monotherapy or in combination with first-generation iSRL. Pegvisomant monotherapy normalizes IGF-I in almost all individuals where the dosage is adequately titrated, effectively negating the need for combined therapy to achieve biochemical disease control.^20,21^ Whether concomitant use with iSRL or cabergoline is a cost-effective approach to use of pegvisomant remains contentious; however, it may be beneficial in the few patients who fail to respond to pegvisomant or show tumour enlargement.^22,23^ First-generation iSRLs, however, remain the most utilized and an essential component of the therapeutic armamentarium in treating patients with acromegaly either as monotherapy or in combination with other medical therapies, with patients frequently remaining on these medications for decades.

## Advent of oral somatostatin receptor ligands

Patients with acromegaly experience chronically impaired well-b eing despite biochemical control of GH and IGF-I .^24^ The factors contributing to this are not completely delineated; however, persistent headaches, joint pain, hypopituitarism, and the long-term medications used to maintain remission, along with the medicalisation that occurs in parallel, are likely contributors. Although iSRL therapy has been the mainstay of medical treatment for acromegaly, satisfaction rates are suboptimal, with only 55.7% of patients in one study reporting being satisfied and 85% favouring an alternative treatment that did not involve injections.^25^ iSRL can result in injection site erythema, discomfort and subcutaneous nodule formation, along with the inconvenience of attending either primary or secondary care medical facilities on a monthly basis for the injection. Some patients also describe ‘escape’ of control of their acromegaly-related symptoms towards the end of the injection cycle.^26^

To try to address some of the limitations of iSRLs, recent drug development has centred on trying to improve the convenience and acceptability of this class of medications for the patient, with the overriding aim of improving the quality of life of these individuals. In this respect, two oral SRL formulations, oral octreotide capsules (OOC) and paltusotine, have undergone efficacy and safety studies.

### Oral octreotide capsules

To enhance the oral absorption of octreotide, OOC have an enteric coating, allowing delivery of the drug to the small intestine where it is absorbed via physiological paracellular pathways, aided by a transient permeability enhancer.^27^ Levels of octreotide achieved with this formulation are not dissimilar to subcutaneously injected octreotide 100 µg in healthy volunteers.^28^ The two seminal studies supporting the efficacy and tolerability of OOC in patients with acromegaly were published in 2015 and 2020.^29,30^ The first of these studies enrolled 155 patients across 37 sites with active acromegaly on a stable iSRL dosage for at least 3 months whilst achieving an age-related IGF-I <1.3 fold the upper limit of normal (ULN) and mean GH <2.5 µg/L.^29^ Patients were switched to OOC at least 4 weeks after the last iSRL at a dose of 20 mg twice per day, the capsules being taken at least 2 h after a meal and the patients fasting for an hour after the medication. The dose was escalated to a maximum of 40 mg twice daily based on IGF-I levels and symptoms assessed every 2 weeks. After the optimal dose was achieved, the patients remained on a fixed dosage up to 7 months (core phase) and were then provided the opportunity to enter a 6-month extension phase. The primary endpoint (the proportion of responders [IGF-I <1.3 ULN and GH <2.5 µg/L] at end of the core phase) was achieved in 65%. Eighty-eight (58%) patients chose to enter the extension phase, with 62% of the intention-to-treat population continuing to be responders at end of the 13 months.^29^ Response to OOC was predicted by the degree of baseline control on iSRLs. Symptom control improved from baseline over the duration of the study period. Adverse events were as expected from the known octreotide safety profile, with 14.8% discontinuing treatment due to these events. A further 16.8% discontinued treatment due to treatment failure. The second study, the phase III CHIASMA OPTIMAL study, randomized 56 patients with active acromegaly on stable iSRL for at least 3 months and IGF-I <1.0 x ULN to either OOC or placebo for 36 weeks.^30^ The initial OOC dose was 20 mg twice daily and titrated to a maximum dose of 40 mg twice daily based on symptoms and to maintain IGF-I levels <1.0 ULN. In the 28 patients randomized to OOC, IGF-I increased from 0.8 x ULN at baseline to 0.97 x ULN at week 36, whereas baseline GH levels of 0.66 µg/L were similar at end of study at 0.60 µg/L. The target of IGF-I <1.0 x ULN was met by 58.2%; however, 75% had an IGF-I <1.1 x ULN.^30^ Seven of the 28 patients randomized to OOC reverted to their normal treatment before the end of the study due to treatment failure (n=5) or adverse events (n=2). Adverse events were as expected for the safety profile of octreotide.^30^

The further phase III multicentre MPOWERED study compared OOC to iSRL in patients who were proven to be responsive to both iSRL and oral octreotide.^31^ During the run-in phase, patients controlled on a stable iSRL dose were switched to OOC and the dosage optimized based on symptoms and IGF-I , aiming to achieve an IGF-I <1.3 x ULN and GH <2.5 µg/L. In line with the previous studies, 64% of the initial cohort maintained the biochemical targets. Thereafter, patients were randomized 3:2 to OOC or their previous dose of iSRL for 36 weeks. At the end of the study, 91% and 100% of those receiving OOC and iSRL, respectively, maintained biochemical response, confirming non-inferiority of OOC compared with iSRL and durability of response. Notably, the OOC arm pre-randomization contained a greater proportion of patients with higher IGF-1 levels, receiving higher iSRL doses pre-randomization, and a greater proportion of patients with magnetic resonance imaging-evident tumour residuum.^31^ Of the patients who received iSRL, 47% reported injection site reactions, of which 81% felt that their daily activities were impacted; however, treatment satisfaction and work productivity were not different between the treatment groups.^31^

In summary, twice-daily OOC appear to maintain control of biochemical markers of disease in at least 60% of patients who had previously achieved biochemical control whilst receiving iSRLs. When patients achieve biochemical control with OOC, the response appears durable in the long term.^32,33^

### Paltusotine

Paltusotine is a potent orally bioavailable non-peptidyl selective SSTR2 agonist being positioned as a treatment for patients with acromegaly and neuroendocrine tumours. In the phase I healthy volunteer study, a single dose of paltusotine led to dose-dependent suppression of GHRH-induced GH. Repeat daily dosing of paltusotine over 7–10 days led to a 19–37% decrease in IGF-1 levels.^34^ Pharmacokinetics from the study estimated the elimination half-l ife at 30 h, consistent with once-daily dosing.

The recently published phase II ACROBAT Edge study provides the first data assessing the safety and efficacy of paltusotine in patients with acromegaly.^35^ Patients were enrolled into the primary analysis cohort and four additional exploratory groups. The primary analysis cohort (Group 1) comprised patients who were partial responders to stable iSRL. Partial responders were defined by an IGF-I value >1.0 x ULN, but <2.5 x ULN during screening visits. The exploratory groups were receiving more intensive medical regimens and included patients receiving iSRL in combination with a dopaminergic agonist who were either partial (Group 2) or complete responders (Group 3; IGF-I <1.0 x ULN); patients receiving pasireotide who were complete responders (Group 4); and patients receiving iSRL in combination with pegvisomant who were complete responders (Group 5). Prior to screening, all patients had been on stable medication for at least 3 months. During the 13-week study, participants underwent double-blinded dose escalations of paltusotine at weeks 2, 5 and 8, dependent on IGF-I levels, symptoms and tolerability. Paltusotine was commenced at a once-d aily dose of 10 mg and titrated to a maximum dose of 40 mg daily to achieve an IGF-I target of <1.0 x ULN. The medication was taken after a 6 h fast with further fasting for 2 h after drug ingestion. A 4-week washout was undertaken at the end of the 13 weeks of active treatment.^35^

Overall, 47 patients were enrolled across the groups, with 25 in the primary analysis cohort. In this sub-cohort, the primary endpoint was change in IGF-I from baseline when the patients were receiving long-acting iSRLs. At the end of the 13 weeks, there was no significant change in IGF-I or GH levels from baseline in this group, with 87% achieving an IGF-I value within 20% of baseline or below. Seventy-eight percent of the cohort were receiving the maximum dose of 40 mg paltusotine daily at week 13. Unsurprisingly, there was a significant increase in GH and IGF-I levels following the paltusotine washout, confirming the patients had active acromegaly. In the pooled cohort receiving both iSRL and dopaminergic agonists (Groups 2 and 3), the median increase in IGF-I and GH was 0.28 x ULN and 0.68 µg/L respectively at 13 weeks, with most patients receiving a paltusotine dose of 40 mg daily. No change in symptom scores occurred across the study, likely reflecting the low symptom burden at baseline. An impression of improvement was reported as ‘very much’ or ‘much’ improved in 23.4% of patients, whilst 55.3% scored themselves as ‘minimally improved’ or ‘no change’. Importantly, no patients reported worsening of their disease, required rescue treatment or had to stop paltusotine due to adverse events. Adverse events were in keeping with those expected from SRL or the disease process itself and no treatment-related serious adverse events were reported.^35^

Therefore, paltusotine shows promise as an alternative to iSRL to control IGF-I and GH levels and thus biochemical control of the disease in a significant proportion of patients maintained on iSRL. The caveat to this is that in contrast to the studies with OOC, which examined the proportion of complete responders to iSRL who maintained a complete response, the only published data for paltusotine is in partial responders to iSRLs. Further studies utilizing paltusotine are, however, in progress (phase III PATHFNDR-1 and PATHFNDR-2 studies [ClinicalTrials.gov identifiers: NCT04837040 and NCT05192382]) and will provide more insight into the efficacy of this novel analogue. Notably the PATHFNDR studies and the extension arm of the ACROBAT Edge (ACROBAT Advance [ClinicalTrials.gov Identifier: NCT04261712]) study are utilizing the new tablet formulation of paltusotine that allows the daily dose to be increased to 60 mg once daily and fasting following ingestion to be reduced to 1 hour.^36^

## Where do we expect to see use of oral somatostatin receptor ligands?

Both OOCs and paltusotine are very welcome adjuncts to the therapeutic armamentarium for management of patients with acromegaly. There is no head-to-head comparison to determine if one of these products is more advantageous compared with the other. The position of oral SRLs (oSRL) within the therapeutic pathway is likely to expand as further data become available from interventional studies and real-world data. To date, there are no published data on prevention of growth of somatotrophinomas or tumour shrinkage; however, it would be intuitive to believe that if the biochemistry is controlled, tumour mass is also likely to be so. The use of iSRL pre-operatively is predicated, at least in part, on the ability of these medications to reduce tumour volume in a significant proportion of individuals with macroadenoma and improve associated co-morbidities. The absence of data with the oSRL relating to tumour shrinkage and improvement in co-morbidities with use of oSRLs would mean that they are not as yet suitable alternatives to iSRL at the pre-operative stage in the management pathway.

Based on the available data, the initial use of the oSRL is likely to be in patients with biochemical control of their disease with iSRL and who wish for an alternative to 4 weekly injections of the current first-generation long-acting iSRL. Based on the data we have available, not all individuals who switch from iSRL to oSRL are likely to achieve biochemical targets.^29,30,35^ Data from the studies of OOC showed that in individuals with controlled disease on iSRL, those with IGF-I and GH values in the lower reaches of the target range are more likely to maintain biochemical disease control with OOC,^29^ enabling clinicians to select patients that oSRL may be most appropriate for.

Although it is hoped that oSRL will cause less disruption for patients, the need to fast before and following oSRL use, may be intrusive on some individual's routines and daily eating habits. In this respect, paltusotine has the advantage over the OOC in being a once-daily medication. Furthermore, the new formulation of paltusotine requires only 1 hour of fasting after being taken. Compliance with oral medication is generally lower when patients are left to their own devices outside of clinical trials. This may be reflected in a lower proportion of patients achieving long-term biochemical control than current interventional trials suggest when real-world data are analyzed in the future.

Many patients with controlled disease on iSRLs will be relieved with the advent of oSRLs. The advantages are clear with regards to the absence of injection site pain, no need for monthly hospital visits for injections and importantly given the shorter half-l ife, the dose can be titrated more rapidly to reach biochemical and symptomatic improvements. This shorter life also has the advantage of allowing patients to cease the medication quicker should side effects be intolerable.
